# Type I Diabetes-Associated Tolerogenic Properties of Interleukin-2

**DOI:** 10.1155/2011/289343

**Published:** 2011-05-10

**Authors:** Aziz Alami Chentoufi, Simon Gaudreau, Alex Nguyen, Mahmoud Sabha, Abdelaziz Amrani, Geyhad ElGhazali

**Affiliations:** ^1^Department of Immunology, Faculty of Medicine, King Fahad Medical City, Riyadh 11 525, P.O. Box 59046, Saudi Arabia; ^2^Immunology Division, Department of Paediatric, Faculty of Medicine and Health Sciences, University of Sherbrooke, Sherbrooke, QC, Canada J1H 5N4; ^3^Faculty of Medicine, Touro University Nevada, Henderson, NV 89014, USA; ^4^Faculty of Medicine, St. George's University, Bay Shore, NY 11706, USA; ^5^Faculty of Medicine and Medical Sciences, University of Shendi, Sudan

## Abstract

Type 1 Diabetes (T1D) results from insulin-producing beta cells destruction by diabetogenic T lymphocytes in humans and nonobese diabetic (NOD) mice. The breakdown of tolerance has been associated with a defect in the number and the function of naturally occurring regulatory T cells (nTreg) that are the master player in peripheral tolerance. Gene knockout experiments in mouse models have shown a nonredundant activity of IL-2 related to its critical role in inducing nTreg and controlling peripheral T cell tolerance. Whereas strong evidence has suggested that IL-2 is critically required for nTreg-mediated T1D control, several fundamental questions remain to be addressed. In this paper, we highlight the recent findings and controversies regarding the tolerogenic properties of IL-2 mediated through nTreg. We further discuss a potential link between the immunomodulatory role of interleukin-2 and the pathogenesis of type 1 diabetes.

## 1. Introduction

The induction of tolerance is critical for the maintenance of immune homeostasis and the prevention of autoimmune diseases, including type 1 diabetes (T1D). Tregs are crucial for suppressing autoimmune responses and maintaining peripheral immunological tolerance [1]. Defects in the number and function of immunoregulatory CD4^+^ T cells (nTregs) play a critical role in the breakdown of immune tolerance in the experimental model of spontaneous autoimmune diabetes nonobese diabetic (NOD) mouse [2, 3] and in humans with genetic susceptibility to T1D. Tregs arise during the normal process of T cell maturation in the thymus, and their differentiation can be induced ( iTreg) in the periphery by conversion of naive CD4^+^CD25^−^Foxp3^−^ Tregs into CD4^+^CD25^+^Foxp3^+^ Tregs [4, 5]. The influence of nTregs in maintaining T cell tolerance is strongly supported by the observations of the development of autoimmune syndromes in mice lacking nTregs and by the findings that defects in Foxp3 gene expression in humans and mice lead to autoimmune syndromes in early life [6, 7]. In agreement with these observations, the prevention of other autoimmune diseases such as rheumatoid arthritis (RA), inflammatory bowel disease (IBD), and type 1 diabetes (T1D) has been achieved by reconstitution of autoimmune-prone mice with nTregs [8]. Emerging evidence has revealed the involvement of IL-2 as a major regulator of the survival and suppressive function of nTreg [9, 10]. Work from Santamaria's group [11] has revealed that IL-2 production was reduced in NOD mice and correlated with impairment in nTreg function. Furthermore, treatment with IL-2 has been shown to induce Treg expansion and activation in humans and mice [9] and protection against diabetes in NOD mice [12]. In the last decade, much progress has been made in understanding the role of the IL-2/IL-2 receptor (IL-2R) axis in promoting nTreg differentiation and its importance in the interface between tolerance and autoimmunity. This paper primarily focuses on our current understanding of the role of IL-2/nTreg in regulating autoimmune diabetes and its potential therapeutic application in patients with T1D. Recently, it has been shown that the administration of low doses of IL-2 at the onset of diabetes can induce a long-lasting remission in NOD mice. Interestingly, IL-2 did not stimulate autoreactive effector T cells but rather specifically stimulated CD4^+^Foxp3^+^ Tregs in the pancreas, resulting in dampening the influence of the inflammatory environment [12]. Here, we further highlight the role of IL-2/IL-2R in autoimmune T1D, specifically through the modulation of nTregs development and function.

## 2. Biological Importance of IL-2/IL-2R Signaling

**Figure 1:**
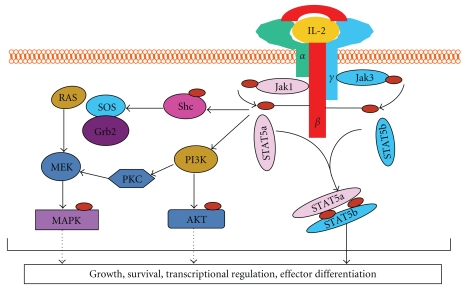
IL-2 receptor signaling. The binding of IL-2 to IL-2R leads to the initiation of signal transduction. Janus-activated kinase 3 (JAK3) molecules that are associated with the *γ*c, and JAK1 molecules that are associated with IL-2R*β*, phosphorylate (the red circle) tyrosine residues in the cytoplasmic tail of IL-2R*β*, the *γ*c and the JAK molecules themselves. The JAKs activation induced a cascade of activation of signal transducer and activator of transcription (STAT5a/b) factors, leading to their dimerization translocation to the nucleus, as well as phosphatidylinositol 3-kinse (PI3K) and ras-mitogen-activated protein kinase (MAPK) signaling pathways. These signaling molecules activation results in, modulation of target genes expression involved in cell cycles progression, antiapoptosis, and in the suppression of cytokine signaling.

IL-2 is a 15 kDa 4-bundled *α*-helical protein mainly produced by activated CD4^+^ T lymphocytes. However, the expression of IL-2 by naïve CD8^+^ T cells, dendritic cells, and thymic cells has also been reported [13, 14]. The magnitude and duration of the T cell immune response is dependent on the interaction of IL-2 with its high-affinity IL-2 receptor (IL-2R) which is composed of *α*, *β* and *γ* subunits. The intermediate affinity IL-2R is composed of IL-2R*β* (CD122) and IL-2R*γ* (CD132) and is constitutively expressed on resting T lymphocytes. However, IL-2R*α* (CD25) is only induced after T-cell activation, which allows the formation of the high-affinity IL-2R [15]. The biological activities resulting from the binding of IL-2 to its receptor on T-cells have not yet been fully defined. Evidence has shown that in conventional T cells, the effects of occupation of the high affinity IL-2R are mediated by at least two major signaling pathways, the JAK-STAT and the PI3K pathways. The activation of the JAK-STAT pathway is initiated by the activation of the Janus Kinases (JAK) JAK1 and JAK3. JAK1 is primarily associated with the serine-rich region of the IL-2R, whereas JAK3 seems to be associated with both the proximal and distal regions of the cytoplasmic domain of the common *γ*-chain. The stimulation of JAK molecules initiates a cascade of activation involving the signal transducers and activators of transcription (STAT5a/b) factors as well as the phosphatidylinositol 3-kinse (PI3K) and ras-mitogen-activated protein kinase (MAPK) signaling pathways [15, 16]. The activation of these signaling molecules results in modulation of target gene expression involved in cell cycle progression (gene encoding cycling family proteins A, B, C, D2, D3, and E), anti-apoptosis (Cmyc, C-fos, C-jun, Bcl2, and bclx), and in the suppression of cytokine (SOCS and CIS) signaling (Figure 1) [17, 18].

The JAK/STAT5 pathway is important for many cellular responses, including differentiation, proliferation, and oncogenesis. For example, STAT5^−/−^mice have a profound defect in mammary gland development and in prolactin response, whereas STAT5B^−/−^ mice display a defect in growth hormone response [19]. Simultaneous inactivation of STAT5A/B genes has revealed a requirement for both proteins in myeloid and lymphoid cell proliferation. Indeed, myeloid cells, mast cells, peripheral T cells, and NK and B cells display impaired proliferation and/or survival in STAT5^−/−^ mice [20–22]. STAT5 has also been shown to be involved in maintaining CD4^+^CD25^+^ regulatory T cell homeostasis and self-tolerance [23], Th2 differentiation, and CD8^+^ T cell homeostasis [24]. STAT5^−/−^ mice have a decreased number of CD8^+^ T cells, whereas STAT5 transgenic mice have an increased number of these cells, which correlates with anti-apoptotic protein Bcl-x_L_ expression [25]. Ectopic expression of Bcl-x rescues STAT5^−/−^ BM cells from apoptosis indicating that STAT5 promotes survival of myeloid progenitor cells through the ability to induce the transcription of the Bcl-x gene [26]. Finally, STAT5^−/−^ mice exhibit autoimmune pathology in a manner very similar to IL-2-deficient mice. This disease correlates with decreased numbers of nTregs, which undergo apoptosis at increased rates in the absence of STAT5 [26]. Recently, it has been reported that a patient, with a missense mutation in the STAT5B gene, had no detectable expression of STAT5B and had decreased numbers and impaired function of nTregs [27]. Similarly, JAK3−/− mice have low frequency of nTregs, increased amount of autoreactive T cells, and develop autoimmunity as is the case for IL-2-deficient and IL-2R-deficient mice [23]. These reports underlie the importance of IL-2/IL-2R signaling in the maintenance of immune tolerance. 

## 3. Naturally Occurring CD4^+^CD25^+^ Tregs and Autoimmunity

Naturally occurring CD4^+^CD25^+^ regulatory T cells represent 5%–10% of the CD4^+^ T cell subset that critically contributes to maintenance of peripheral immune tolerance. These cells arise in the thymus and, after migrating to the periphery, exert their immunoregulatory functions. They are potent suppressor of organ-specific autoimmune diseases such as T1D, inflammatory bowel disease and gastritis, and they control allograft rejection and immunity to infectious agents such as parasites and viruses [28–30]. In mice, a day-3 neonatal thymectomy (d3Tx) leads to the development of multiorgan autoimmune disease [31]. Subsequently, the depletion of CD4^+^CD25^+^ Tregs leads to spontaneous development of various autoimmune diseases in genetically susceptible animals as well. The adoptive transfer of CD4^+^CD25^+^ nTreg prevents the development of such organ-specific autoimmunity [32]. In the T1D NOD mouse model, it has been shown that the pool of CD4^+^CD25^+^ regulatory T cells decreases with the progression of diabetes [33, 34] and that administration of these cells affords protection against the development of diabetes [35, 36]. In the mouse, nTreg are considered as resting antigen-experienced cells and display heterogeneous combination of cell surface markers associated with naive and activated T cells. nTregs constitutively express high-affinity IL-2R*α* (CD25) and Foxp3, a forkhead winged helix transcriptional regulator that controls their development and functions [37]. The mutation of Foxp3 resulted in drastic loss of nTregs and fatal lymphoproliferative process that leads to multiorgan-autoimmune diseases of nTregs in mice and humans. The transfer of nTreg cells from wild-type mice to scurfy mice rescues the animals from the fatal disease [7, 38]. Other cell surface markers have been shown to be expressed by nTregs, including high levels of CD5, CD62L, and CD69. Beside the expression of high-affinity IL-2R*α* (CD25), at resting state, nTreg constitutively express high levels of other feature markers such as the glucocorticoid-induced TNFR (GITR) family of related proteins [39], OX40 (CD134) [40, 41], and CTLA-4 [42, 43] that also contribute to their suppressive function. Importantly, mice deficient in Foxp3 gene (scurfy mice) rapidly develop a fatal lymphoproliferative disease similar to that seen in mice lacking CTLA-4 or TGF-*β* [44]. Recently, it has been shown that OX40 is a key factor in shaping nTregs sensitivty to IL-2 and promoting their proliferation and survival toward accurate immune regulation [45]. 

We have previously reported that tolerogenic DCs are critical in maintaining nTregs pool in NOD mice. Using adoptive transfer experiments, we have shown that depletion of tolerogenic DCs before transferring splenocytes from diabetes-free NOD mice restored their diabetogenic potential, thereby underlying the importance of tolerogenic DC in nTregs maintenance [46]. Naïve CD4^+^CD25^−^ T cells can be also converted into regulatory-like T cells after transduction with a retroviral vector coding for the FoxP3 gene. The transfer of these converted Tregs has been shown to prevent the development of autoimmune IBD [5, 38, 47]. Collectively, these data clearly indicate that Foxp3 plays a critical role for both development and function of nTreg in mice.

## 4. Cytokines Profile and Cytokines Receptor Signaling in nTreg

The mechanisms by which IL-2 or other cytokines exert their effects via the cytokine receptors on nTreg have not been fully investigated yet. In conventional T cells, the effects of IL-2 are mediated by at least two major signaling pathways, the JAK-STAT and PI3K pathways. Several different studies have examined the role of JAK-STAT molecules in CD4^+^CD25^+^ nTreg development and function. The importance of JAK3 in IL-2 signaling has been further substantiated by the recent findings that homozygous point mutations or deletions of the Jak3 gene are found in autosomal recessive T-B+ severe combined immunodeficiency patients [48] as well as immunocompromised mice [49]. Indeed, mice deficient in JAK3 or STAT5A/B develop a severe immunodeficiency that is followed by multiple organ autoimmune diseases, a decreased number or the absence of CD4^+^CD25^+^ nTreg in their peripheral lymphoid organs, and early death. However, these mice have normal numbers of Tregs in the thymus, suggesting the role of these molecules in regulatory T cell development and homeostasis [23, 26]. Interestingly, the overexpression of STAT5b in transgenic mice leads to an increased number of CD4^+^CD25^+^ nTreg [50]. 

The role of JAK-STAT and PI3K signaling pathways in Treg proliferation and immunoregulatory function is not yet fully defined. Recently, Bensinger et al. [51] have shown that freshly isolated CD4^+^CD25^+^ nTreg maintain an intact JAK-STAT signaling pathway, whereas signaling downstream PI3K (particularly activation of AKT and p70S6K) is negatively regulated as a result of increased expression of phosphatase and a tensin homolog deleted on chromosome 10 (PTEN). Conversely, we found that *in vitro* stimulated Treg displayed a normal PI3K pathway while inhibiting JAK-STAT pathways, which results from a downregulation of JAK-STAT molecules expression as opposed to kinase activity (Chentoufi et al. Unpublished data). The inhibition of the JAK-STAT pathway could result from the high expression of SOCS-2 and CIS molecules observed 24–48 h following nTreg stimulation with anti-CD3 and IL-2. These results may explain the hypoproliferative activity of regulatory T cells even after activation through TCR in the presence of IL-2. Moreover, IL-2 generates signaling pathways through a cytokine receptor that contains a *γ*-chain common to several other cytokine receptors including IL-4, IL-7, IL-9, IL-15, and IL-21. These observations add more complexity to the problem of deciphering the role of other cytokines on CD4^+^CD25^+^ nTreg. 

Recently, it has been reported that IL-4 can substitute for IL-2 to induce CD4^+^CD25^+^ nTreg-mediated proliferation and suppression *in vitro*. However, nTreg obtained from IL-4−/− mice display normal suppressive activity, suggesting profound differences between *in vitro *and *in vivo *effects [52]. It has also been shown that STAT1-deficient mice are highly susceptible to autoimmune diseases resulting from a reduced number as well as a functional impairment of CD4^+^CD25^+^ nTreg, suggesting a role of IFNs on regulatory T cells [53]. Naturally occurring CD4^+^CD25^+^ T cells constitutively express mRNA coding for IL-10, IL-4, IL-17, IL-21, IFN-*γ*, TNF-*α*, and TGF-*β* but do not express mRNA coding for IL-2 [43].

The biological roles of cytokines production and interaction with their receptors in nTreg development and function have not yet been clearly delineated. Recently, McHugh et al. [43] have shown, through gene expression profiling experiments, that JAK-STAT regulatory proteins such as members of the SOCS family of proteins (SOCS-1, SOCS-3, and CIS) are upregulated in stimulated CD4^+^CD25^+^ nTreg as compared to conventional T cells [43]. The hypoproliferative activity of activated nTreg *in vitro* and the increased expression of SOCS molecules could suggest that these molecules play a role as an internal control mechanism for CD4^+^CD25^+^ nTreg activation and expansion. SOCS molecules function to inhibit the proliferative effects of cytokines such as IFN-*γ*, IL-4, and IL-2 and can antagonize JAK3 and STAT5 activities [18].

## 5. Critical Balance of IL-2 for Naturally Occurring CD4^+^CD25^+^ Treg-Mediated T1D Control

IL-2 was originally defined as a growth factor that stimulated the differentiation and proliferation of T lymphocytes [54]. The critical role of IL-2 on the survival and suppressive function of nTreg cells has been well documented [55]. Consistent with this, IL-2 administration has been shown to expand and activate nTreg cells in humans and mice [9, 10]. Thus, although IL-2 has pleiotropic functions, its major impact is to favor nTreg cell activity [56]. Surprisingly, in IL-2 or IL-2R knockout mice, T cells develop, and these mice acquire a lymphoproliferative syndrome and spontaneous autoimmune disease [57]. From these studies, two important roles for IL-2 in immune tolerance can be ascribed. First, the data showed that IL-2 played a critical role in programming T cells for activation-induced cell death (AICD). Second, increased evidence showed that IL-2 played an additional role in immune regulation aside from AICD. For instance, several groups have reported that in IL-2 and IL-2R knockout mice, the lymphoproliferative syndrome could be prevented by rIL-2 injection and bone marrow transplantation from wild-type mice [58]. 

Of importance, it was observed that IL-2 had to be provided at very early stages. For instance, delivery of IL-2 a few days after birth resulted in low efficiency of disease improvement, suggesting that it was not the lack of IL-2 during later T cell activation in the periphery that was at fault in these mice. The dependence of nTreg cells on IL-2 was first suggested by transfer experiments performed by Klebb et al. These authors transferred spleen cells or thymocytes from IL-2−/− mice treated with rIL-2 for 25–35 days into untreated 6–8-day-old IL-2−/− BALB/C mice. The IL-2 defect on the BALB/C background is particularly severe and leads to death within 3 weeks. Depending on the number of transferred cells and the organ from which they were derived, some of the recipients lived for 7–10 months. Since no more IL-2 was available during that time, the authors suggested that “IL-2 induces a postnatal differentiation/maturation of regulatory cells necessary for self and non-self-discrimination”. Kramer et al. have shown that IL-2−/− and IL-2R−/− bone marrow transfer into RAG−/− mice leads to the development of IL-2 deficiency syndrome, and early death of RAG−/− recipient mice [59].

Again, the disease could be prevented by the complementation with bone marrow from IL-2-sufficient mice. Transfer of CD4^+^CD25^+^ nTreg from wild-type mice induced the development of a lymphoproliferative syndrome in IL-2R*α*−/− mice [60]. The most striking observation in IL-2−/− mice models is the lack of CD4^+^ CD25^+^ nTreg, suggesting a critical role for IL-2/IL-2R signaling in the generation and homeostasis of these cells. Indeed, mice deficient in IL-2, IL-2R [61], JAK-3 [23], or STAT5A/B [26] have an absent or reduced number of thymic and peripheral CD4^+^ CD25^+^ nTreg and consequently develop multiple organ autoimmune diseases. To evaluate the role of IL-2 expression on thymic nTreg development, Malek et al. rescued IL-2R*β* knockout mice with an IL-2R*β* transgene that was predominantly expressed in the thymus with negligible expression in the periphery [60, 61]. In marked contrast to IL-2R*β*−/− mice, the IL-2R*β* transgene restored the normal levels of CD4^+^CD25^+^ nTreg and prevented the onset of a fatal autoimmune disease, further reinforcing the notion that IL-2 plays a critical role in the thymic development of nTreg. 

The molecular mechanism by which IL-2 exerts its effect on the nTreg cell precursors in the thymus is as of yet not completely understood. In addition, the origin of IL-2 in the thymus does not seem to be from CD4^+^CD25^+^ nTreg themselves or hematopoietic cells, but probably derives from radio-resistant thymic stromal cells. Indeed, chimeric bone marrow from IL-2−/− and IL-2R*α*−/− mice develops functional CD4^+^CD25^+^ nTreg. Furthermore, lethally irradiated RAG2−/− mice that have been reconstituted with IL-2−/− bone morrow develop normal CD4^+^CD25^+^ nTreg [62]. The IL- 2 signaling requirement for peripheral expansion/homeostasis of CD4^+^CD25^+^ nTreg has been shown by Malek et al. when CD4^+^CD25^+^ nTreg from wild-type mice were adoptively transferred to IL-2R deficient mice (which normally produce IL-2). The recipient mice did not develop autoimmune diseases. In contrast, the adoptive transfer of CD4^+^CD25^+^ nTreg from wild-type mice failed to confer the same benefits in IL-2−/− mice [61]. 

The requirement of IL-2 for nTreg suppression activity is still very controversial. When nTreg are adoptively transferred, they lose CD25 expression and hence are no longer responsive to IL-2. Nevertheless, these cells retain their suppressive activity *in vitro* [63]. However, the addition of IL-2 to nTreg eliminates this suppressive activity *in vitro* [64]. Conversely, CD4^+^CD25^+^ nTreg from wild-type mice transferred to IL-2-deficient mice fail to prevent autoimmunity and *in vitro* suppression activity is completely abrogated by selective blocking of the IL-2 receptor on nTreg during a coculture with responder T cells [65]. Furthermore, treatment of mice with anti-IL-2 or with CTLA-4 immunoglobulin to inhibit costimulatory signals also leads to a rapid decline in the number of CD4^+^CD25^+^ T cells [36]. 

Besides, NOD mice present a qualitative diminution of IL-2 production [11], and a genetic predisposing factor to T1D development in humans and NOD mice is linked to IL-2/IL-2R gene polymorphisms [66]. It has been recently reported that insufficient IL-2 amounts in the pancreas are responsible for poor nTreg cell survival in this tissue, which could lead to progressive breakdown of self-tolerance and the development of diabetes in NOD mice [67]. It has been recently shown that young prediabetic NOD mice treated with low-dose IL-2 alone, or together with rapamycin, can be protected from the development of disease [67–69]. Recently, it has been shown that low-dose IL-2 administration at diabetes onset can induce long-lasting remission in the treated mice. Interestingly, IL-2 seems to specifically stimulate the CD4^+^CD25^+^ Treg cells in the pancreas rather diabetogenic effector T cells [12]. The mechanisms by which IL-2 selectively stimulate Tregs and reverse the disease in NOD diabetic mice is not yet understood. One mechanism of action of low-dose IL-2 would be the limitation of IFN-*γ* production by the islet-infiltrating effector T cells. In addition, by expressing high levels of the high affinity IL-2R, Tregs have an advantage in competing for low levels of IL-2 against effector T-cells in the microenvironment. 

A number of contradictory observations regarding the number and function of nTreg in diabetic individuals and in mice have been reported. Regulatory T cell numbers have been reported to be decreased or normal in T1D [70–79], and functional assays have similarly described low, slightly decreased, or normal regulatory activity [80–86]. Nevertheless, a consensus regarding a few key points is developing. (i) There is clear evidence for the existence of natural regulatory T cells in both pre-T1D and postdiagnosis T1D subjects and mice. (ii) Some of the induced Treg cells display similar antigen specificity for *β*-cell autoantigens of non-T1D subject's Tregs [82]. (iii) In vitro and in vivo expansion of nTreg cells have been successful both in mice and human, particularly aided by the use of rapamycin [87, 88].

## 6. Conclusion and Perspectives

**Figure 2:**
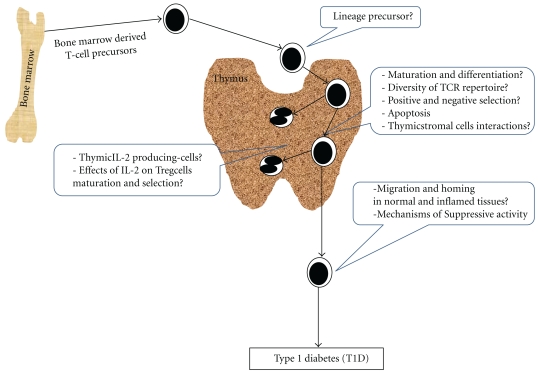
Fundamental questions about naturally occurring CD4^+^ CD25^+^ regulatory T cells development and function.

It has now been strongly established that T cell-mediated dominant immunoregulation is essential for maintaining immunologic self-tolerance and controlling immune responses to non-self-antigens. Among the various kinds of Tregs (iTreg, Tr1, and Th3), naturally occurring CD4^+^CD25^+^ nTreg play a key role in peripheral self-tolerance. A proteomic approach might contribute better to the identification of novel molecules involved in IL-2R signaling pathways for further therapeutic methods and specific cell surface markers for better isolation and characterization purposes. *In vivo*, if IL-2 seems to play a critical role in the thymic development of nTreg, which cells produce IL-2 in the thymus, at which stage of T-cell development IL-2 exert its effects, and what is the importance of IL-2 for the nTreg precursor cells differentiation are critical questions that need to be investigated. A possible explanation is that some thymocytes are confined within special thymic microenvironments containing high doses of IL-2, which could, in principle, transform them into anergic Treg cells. Such an anergic state could be obtained *in vitro *following Th1 clones incubation with IL-2 for 24 to 48 h. These cells are rendered unresponsive to subsequent restimulation with antigen [89] although they remain fully responsive to IL-2 *in vitro *as long as IL-2 is provided. However, when restimulated with appropriate antigen and APC, they do not secrete cytokines, including IL-2 [90]. With respect to T cell maturation and central tolerance, not much is known about nTreg cells thymic maturation/differentiation. Whereas it is thought that nTreg cell generation depends on self-antigen expression in the thymus [91], a recent report from the Mathis' group [92] has shown that nTreg does not necessarily require the thymic expression of agonistic self-antigen. These questions and more need to be further investigated. Certainly, the development of technical tools and animal models will be invaluable to shed light into naturally occurring Treg biology and to provide novel immunomodulatory approaches that target (i) the downregulation of the immune system to prevent/treat autoimmune disease and allograft rejection and (ii) the control of immunity to infectious agents such as parasites and viruses (Figure 2).

## Abbreviations

T1D: Type 1 diabetes
nTreg: Naturally occurring regulatory T-cells
IL-2: Interleukin-2.
